# Web-Based Just-in-Time Information and Feedback on Antibiotic Use for Village Doctors in Rural Anhui, China: Randomized Controlled Trial

**DOI:** 10.2196/jmir.8922

**Published:** 2018-02-14

**Authors:** XingRong Shen, Manman Lu, Rui Feng, Jing Cheng, Jing Chai, Maomao Xie, Xuemeng Dong, Tao Jiang, Debin Wang

**Affiliations:** ^1^ School of Health Service Management Anhui Medical University Hefei China; ^2^ Department of Literature Review and Analysis Library of Anhui Medical University Hefei China

**Keywords:** internet, drug resistance, bacterial, primary health care, randomized controlled trial, China

## Abstract

**Background:**

Excessive use of antibiotics is very common worldwide, especially in rural China; various measures that have been used in curbing the problem have shown only marginal effects.

**Objective:**

The objective of this study was to test an innovative intervention that provided just-in-time information and feedback (JITIF) to village doctors on care of common infectious diseases.

**Methods:**

The information component of JITIF consisted of a set of theory or evidence-based ingredients, including operation guideline, public commitment, and takeaway information, whereas the feedback component tells each participating doctor about his or her performance scores and percentages of antibiotic prescriptions. These ingredients were incorporated together in a synergetic way via a Web-based aid. Evaluation of JITIF adopted a randomized controlled trial design involving 24 village clinics randomized into equal control and intervention arms. Measures used included changes between baseline and endpoint (1 year after baseline) in terms of: percentages of patients with symptomatic respiratory or gastrointestinal tract infections (RTIs or GTIs) being prescribed antibiotics, delivery of essential service procedures, and patients’ beliefs and knowledge about antibiotics and infection prevention. Two researchers worked as a group in collecting the data at each site clinic. One performed nonparticipative observation of the service process, while the other performed structured exit interviews about patients’ beliefs and knowledge. Data analysis comprised mainly of: (1) descriptive estimations of beliefs or knowledge, practice of indicative procedures, and use of antibiotics at baseline and endpoint for intervention and control groups and (2) chi-square tests for the differences between these groups.

**Results:**

A total of 1048 patients completed the evaluation, including 532 at baseline (intervention=269, control=263) and 516 at endpoint (intervention=262, control=254). Patients diagnosed with RTIs and GTIs accounted for 76.5% (407/532) and 23.5% (125/352), respectively, at baseline and 80.8% (417/532) and 19.2% (99/532) at endpoint. JITIF resulted in substantial improvement in delivery of essential service procedures (2.6%-24.8% at baseline on both arms and at endpoint on the control arm vs 88.5%-95.0% at endpoint on the intervention arm, *P*<.001), beliefs favoring rational antibiotics use (11.5%-39.8% at baseline on both arms and at endpoint on the control arm vs 19.8%-62.6% at endpoint on the intervention arm, *P*<.001) and knowledge about side effects of antibiotics (35.7% on the control arm vs 73.7% on the intervention arm, *P*<.001), measures for managing or preventing RTIs (39.1% vs 66.7%, *P*=.02), and measures for managing or preventing GTIs (46.8% vs 69.2%, *P*<.001). It also reduced antibiotics prescription (from 88.8%-62.3%, *P*<.001), and this decrease was consistent for RTIs (87.1% vs 64.3%, *P*<.001) and GTIs (94.7% vs 52.4%, *P*<.001).

**Conclusions:**

JITIF is effective in controlling antibiotics prescription at least in the short term and may provide a low-cost and sustainable solution to the widespread excessive use of antibiotics in rural China.

## Introduction

### Background

Antibiotics resistance (ABR) has become an urgent and worldwide public health problem. According to an independent review commissioned by UK Prime Minister David Cameron, around 700,000 people died in 2014 due to antimicrobial resistance and will reach 10 million lives a year by 2050, and a cumulative US $100 trillion of economic output are at risk due to drug-resistant infections if we do not find proactive solutions now [1]. Primary care ranks top priority for curbing the problem since it incurs the majority of prescribed antibiotics [2]. Over 80% of all antibiotics are prescribed in primary care, and there is a wide variation both within and between countries in antibiotic prescribing rates that cannot be explained by differences in the epidemiology of infections [3-5]. The situation of ABR in China, especially rural areas, is among the most serious in the world. A cross-sectional study of 40 counties in rural western China reported that 48.43% of all service episodes at village clinics used at least one kind of antibiotic [6]. Another study conducted in Shandong province revealed that the antibiotics were used for 59.01% of all the patients at village clinics [7].

A variety of measures have been used in improving antibiotics use (ABU) at primary settings. These include education of doctors and patients, structural changes, commitment letters, guidelines, prescription formularies, negative disease lists, shared decision-making (SDM), and others [8-11]. Most of these approaches have showed marginal to moderate effects in various countries. ABR has also gained high recognition in China. Starting from 2009, the then Ministry of Health had launched a nationwide Special Antimicrobial Use Rectification Program. It consisted of a package of interventions, for example, introduction of stewardships, establishment of microbiological laboratories in county or higher-level hospitals, implementation of periodic ABU audits, and capping overall proportion of antibiotic prescriptions, etc. China’s new health reforms also include various systemic changes aimed at curbing excessive ABU, for example, differential lists of antibiotics for different level of health care providers and zero profit from antibiotics prescriptions [12,13]. Although these efforts have been associated with reductions in antibiotic prescribing at county or higher-level public hospitals, there are indications that antibiotic use at primary care settings remains very high [14]. This is especially true in resource-poor rural areas where more than 60% of the nation’s vast population resides [15].

### Objectives

In response to the above challenges and others, this study aimed at developing and evaluating an innovative intervention, which provides the village doctors with just-in-time information and feedback (JITIF). Guided by proven theories and best practices, JITIF incorporates a number of cost-effective and sustainable approaches in a synergetic way in leveraging accountable ABU within routine primary care in rural China. Being designed as a potential package to be routinely used at rural primary care settings, JITIF covers all kinds of infections categorized as respiratory tract infections (RTIs), gastrointestinal tract infections (GTIs), urinary tract infections (UTIs), skin infections (SIs), and eye and dental infections (EDIs). Given that the prevalence of different infections varies greatly, we decided to test and promote JITIF in two stages. The first stage aims at testing its efficacy through a relatively small-scale randomized controlled trial focusing only on the two most common kinds of infections (ie, RTIs and GTIs), whereas the second leverages the experiences and findings from the first into a larger and more comprehensive trial covering all the aforementioned infections. This paper documents the main ingredients and mechanisms of the intervention package and preliminary findings about its efficacy from the first stage study.

## Methods

### Ingredients of Just-in-Time Information and Feedback

As indicated by its title, the intervention comprises two components: information and feedback. The information component consisted of a set of theory and evidence-based ingredients, including operation guidelines, public commitment, and takeaway information. The operation guidelines divided routine care of symptomatic infection patients into 5 pragmatic steps (patient categorization, disease diagnosis, SDM, drug prescription, and patient education) and provided standard operation procedures (SOPs) for each of them.

The public commitment asked each of the participating village doctors in the intervention group to sign a letter of commitment and made the signed letter public by posting it on the walls of his or her clinic and printing it on the back of the patient takeaway information leaflet. The letter was first drafted by a researcher and then revised by all the intervention group doctors via a consensus session scheduled at the end of the project orientation training workshop. The finalized letter (see Multimedia Appendix 1) conveyed a clear message that the doctor who had signed the letter was strongly committed to use antibiotics accountably.

The takeaway information (see Multimedia Appendix 2) was divided into two parts and printed on the front and back pages, respectively, on an A4-sized paper for patients to take home. The front page was patient specific and contained mainly: (1) the name of the patient and date of the service encounter; (2) main symptoms, related history (if applicable), and clinical diagnosis of the current infection; (3) whether antibiotics were used and why (including benefits and side effects of ABU); (4) (if applicable) name, dosage, duration, and administration mode of the antibiotics used; (5) (if applicable) agreed plan for symptom relief; (6) (if applicable) agreed plan for backup antibiotics treatment; and (7) suggested activities for preventing the same infection in the future. The back page, however, was mostly the same to all the patients. It contained the commitment letter and a signature of the attending doctor.

The feedback component of JITIF told each participating doctor about his or her performance scores (PSs) and percentages of prescribed ABU. The PSs for any individual doctor were based on the records of his or her management of symptomatic infection patients in the past 3 months and were rated by a panel of experts on care of infectious diseases according to a preset checklist. The percentages of prescribed ABU were also based on the same records and for the same time period but calculated automatically by the Web-based support system. Any PS (or percentage of ABU) for a given doctor was presented in red, yellow, and green, respectively, if it fell below (or above), within, and above (or below) the interquartile range of the same PS (or percentage of ABU) for all the participating doctors assessed in the same time period.

### Implementation of Just-in-Time Information and Feedback

JITIF strived to deliver the abovementioned feedback and information at a time when the recipients were most ready to accept or act upon it. In reaching this end, it used a Web-based aid (WBA) consisting of 5 consecutive pages, each facilitated one of the 5 steps of routine service for infection patients mentioned above. A typical WBA page (see Multimedia Appendix 3) consisted of 2 main columns (the right and the left column). The right column presented SOP for the doctor to follow and is hereafter referred to as the SOP column. The left column was further divided into top and bottom parts used to display relevant performance and references, respectively, and thus referred to as performance and reference column, respectively.

Page 1 (see Multimedia Appendix 3) was used to categorize the patient. Its SOP column presented: (1) 5 structured or open questions followed by input boxes, radio buttons, or dropdown lists designed to remind and facilitate the attending doctor in enquiring and recording the patient’s name, gender, identification number, and address; (2) 1 open question asking about the patient’s main health problems, followed by a list of tick boxes designed to help the doctor in rapidly grouping the patient into specific categories, that is, RTIs, GTIs, UTIs, SIs, EDIs, and noninfectious diseases; (3) 1 structured question asking about whether it was the first visit for the current infection or not. These categorizations lead to different sets of content and SOPs to be addressed in the subsequent pages. The performance column of Page 1 displayed the overall PSs in total and by different type of infections (eg, RTIs, GTIs, UTIs) for the current doctor and his or her peers, whereas the reference column of Page 1 showed the bulleted points of the doctor’s public commitment.

Page 2 dealt with disease diagnosis. Its content varied depending on the category ticked in the previous page. Taking the example of RTIs, the SOP column of Page 2 proposed 3 substeps (SOP_2-1_ to SOP_2-3_). SOP_2-1_ examined common symptoms and signs of RTIs, with particular attention being paid on symptoms and symptom features helpful in distinguishing viral versus bacterial infections. SOP_2-2_ inquired previous formal and informal care for the current illness, especially ABU. SOP_2-3_ reached a clinical diagnosis of RTIs, for example, influenza, sore throat, and common cold. The performance column of Page 2 presented the doctor’s PSs in total and in terms of data completeness and/or accuracy for each of the 3 substeps included in the SOP column, whereas the reference column of Page 2 presented bulleted tips on soliciting information about RTI-related symptoms or signs, history, and distinguishing specific RTIs.

Page 3 leveraged SDM. It stressed adequate discussion between the attending doctor and attendee patient before prescribing any antibiotics. Its SOP column comprised 4 substeps. SOP_3-1_ predicted pathogen and occurrence and trajectory of patient’s symptoms. SOP_3-2_ asked the patient’s worries about the infection and expectations from the visit. SOP_3-3_ enumerated potential treatment options, including vacuum treatment (letting the patient go without prescribing any medicine), symptom relief treatment (carefully selected nonantibiotic alternative measures, for example, traditional food and physical therapies), backup antibiotic treatment and antibiotic treatment, and discussed benefits and side effects of each of them. SOP_3-4_ helped the patient in choosing one of the treatment options discussed. The performance column of Page 3 presented the doctor’s PS on this whole stage and on each of the 4 substeps mentioned above, whereas the reference column of Page 3 presented bulleted indications for using and not using antibiotics for the current type of infection (eg, RTIs, GTIs, and UTIs) and tips on practicing SDM.

Page 4 facilitated prescription if the treatment options chosen during the previous SDM contained antibiotic and/or nonantibiotic medications. Its SOP column provided prescription formularies, and its performance column provided percentage of antibiotic prescriptions by the attending doctor, as compared with that by his or her peers, for patients with different common infections, whereas its reference column provided bulleted rules for choosing medicines for the specific infection under concern (eg, common cold, influenza).

Page 5 promoted patient education. Its SOP column proposed 3 substeps. The content of SOP5_5-1_ depended again on the treatment options chosen during the previous SDM. For options with antibiotic and nonantibiotic medications, it educated use of the medicines prescribed. For vacuum treatment, it proposed carefully selected alternative measures. For backup antibiotic treatment, it informed the patient when and on what indications he or she should come back to the doctor again. SOP_5-2_ counseled regarding the prevention of secondary infection to the patient’s relatives and future reinfection for the patient himself or herself. SOP5_5-3_ developed and printed a tailored takeaway information sheet for the patient. The performance column of Page 5 showed PSs on this whole stage and on each of the 3 activities, whereas the reference column of Page 5 showed bulleted tips on counseling use of medications, backup treatment, and infection prevention.

### Evaluation of Just-in-Time Information and Feedback

Evaluation of JITIF adopted a randomized controlled trial design. The study settings included 12 intervention and 12 control villages in Anhui, a province located in east central China with a population of 68.6 million, of whom 57% live in rural areas. Per capita GDP and income in Anhui rank in the midrange (14 out of 31) among all provinces in China, and the social, cultural, and economic background in Anhui is representative of over 80% of the population in the nation. The study sites were determined via a 3-step clustered randomization. Step 1 divided all the 55 rural counties in Anhui province into north (17 counties), middle (16), and south (22). Step 2 randomly selected: (1) 4 counties from each of the regions, (2) 1 township from each of the selected counties, and (3) 2 administrative villages from each of the selected townships. Step 3 randomly assigned the 2 villages within each township into intervention and control arms. All the 24 village clinics in the selected villages agreed to participate. The 12 clinics on the intervention arm completed a baseline (from August 20 to September 30, 2015) and an endpoint (from August 22 to September 30, 2016) evaluation and implemented JITIF right after the baseline evaluation and throughout the whole study period, whereas the 12 clinics on the control arm completed only the baseline and endpoint evaluation. The study was not registered since randomization was applied only to clinics and not to patients.

Measures used in evaluating JITIF included changes between baseline and endpoint and between control and intervention groups in terms of: (1) percentages of patients with symptomatic RTIs or GTIs being prescribed with oral, intravenous, and injection antibiotics; (2) delivery of essential service procedures, including checking body signs, measuring temperature, performing auscultation, discussing nonantibiotic therapies, educating drug administration, counseling infection prevention, etc; and (3) patients’ knowledge about side effects of antibiotics and infection management and prevention. To enable examining compatibility between baseline versus endpoint and intervention versus control groups, the evaluation also collected data about patients’ age, sex, education, and clinical diagnosis.

Collection of the above data adopted nonparticipative observation using a structured form and face-to-face interview using a structured questionnaire. The observation form (see Multimedia Appendix 4) was designed to solicit data about delivery of service procedures, clinical diagnosis by the doctor, and prescribed antibiotics from patient-doctor encounters happened at village clinics, whereas the structured questionnaire was designed to collect data on knowledge about infection, antibiotics, and infection prevention from the same patients observed. One researcher and one graduate student on health services worked as a group in performing the observation and interview at a clinic, respectively. The observation covered the whole process of eligible patient-doctor encounters, while the interview happened just before the patient had finished his or her consultation and was about to leave the clinic. A total of 4 groups participated in the data collection, each responsible for 3 interventions and 3 control clinics. None of these field data collectors had any relationship that may bias the data collection. Both the baseline and endpoint data collection at each clinic lasted for 1 week. Within the week, all eligible patients were invited to the clinic to participate. The eligibility criteria were men and women who: (1) were 18 years and older; (2) came to the clinic for symptomatic RTI or GTI as the primary health problem; and (3) were willing to participate and able to respond (eg, without mental problems).

The study took a combination of measures in ensuring data quality. These included: (1) training of field observers and interviewers on potential biases and measures avoiding them; (2) clarification of study purpose stressing that both positive and negative findings are of equal interest; and (3) feedback of rating-rerating discrepancies. Here, the feedback consisted of the following: (1) all the patient-doctor encounters were, after informed consent, audio-recorded; (2) a data quality supervisor randomly selected one case of the audio recordings from each of the site clinics every day, concealed all the labeling information, except a unique reference number of the recordings and then sent them to a third researcher; (3) the third researcher rated all applicable essential service procedures according to the audio recordings and using the same rating form as that used by the field observers and then sent the ratings back to the data quality supervisor; (4) the data quality supervisor calculated the differences between the ratings for the encounters with the same reference number but by different generators (one of the field observer and the third independent researcher); and (5) the data quality supervisor sent a short report about the average and observer-specific differences to each of the field observers every day during the data collection period.

### Data Analysis

The completed observation forms and questionnaires were double-entered into a database using EPI DATA (The EpiData Association. Version 3.1) and then analyzed using SPSS (IBM Corporation. Version 11) and Microsoft Excel (Microsoft Corporation. Version 2013). The data analysis consisted mainly of: (1) distribution of respondents and RTIs or GTIs by sociodemographics; (2) estimations of knowledge, delivery of indicative procedures, and use of antibiotics at baseline and endpoint and for intervention and control groups; and (3) chi-square power tests for differences in these estimations between different groups. For additional information about methods, please refer to Multimedia Appendix 5.

### Human Subject Protection

This study involved recruitment, intervention, and assessment of patients and village doctors. So it adhered to rigorous human subject protection principles and procedures. The study protocol had been reviewed and approved by the Biomedical Ethics Committee of Anhui Medical University before study commencement. Participation of villagers and village doctors was voluntary. Written informed consent was sought from all participants.

## Results

### Patient and Doctor Participants

**Figure 1:**
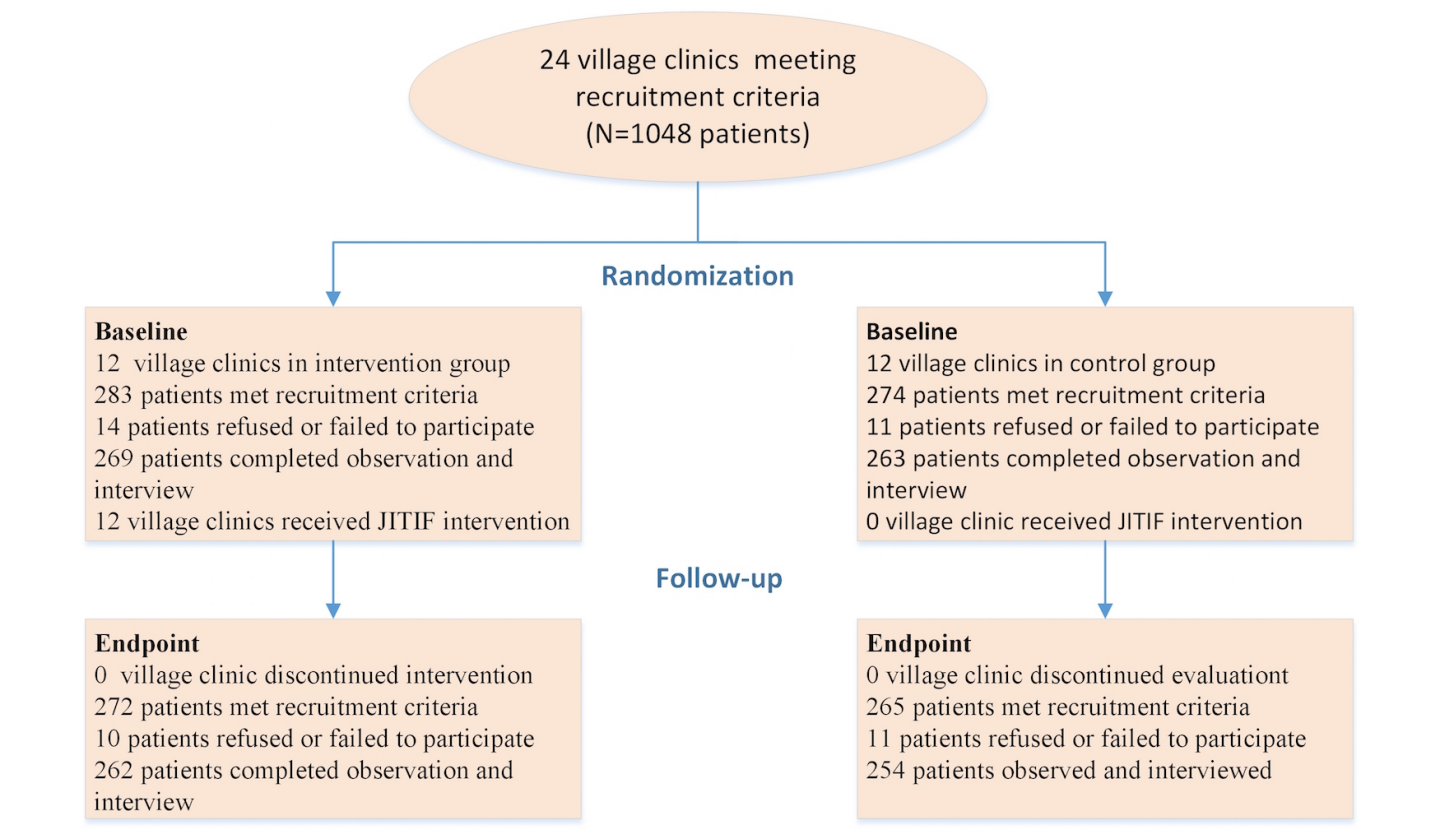
Flowchart of participant selection and follow-up.

As shown in Figure 1, the study recruited 24 village clinics. These clinics had 65 village doctors (2-4 each). These doctors consisted of 23 females and 42 males. They were aged between 32 and 68 years and had been a village doctor for 7 to 58 years. All of them participated in the study without withdrawal. No statistically significant differences were found between the control and intervention group doctors in terms of gender composition and years of age and practice. The overall and item-specific rating-rerating discrepancies of the essential service procedures also did not show any statistically significant differences between the two arms.

Table 1 summarizes characteristics of the participating patients, and Figure 1 shows the flowchart of participant selection and follow-up. A total of 1048 patients completed the observation and interview, including 532 patients at baseline (intervention=269 out of 284, control= 263 out of 274) and 516 patients at endpoint (intervention=262 out of 272, control=254 out of 265). The number of females was more than twice that of males (718 vs 330). The majority of participants had lower than high school education. There was no statistically significant difference in the patients’ age, gender, and education between baseline and follow-up and between control and intervention group (*P*=.42 to .999). Patients diagnosed (by the participating village doctor) with RTIs and GTIs accounted for 76.5% (407/532) and 23.5% (125/532), respectively, at baseline and 80.8% (417/532) and 19.2% (99/532) at endpoint.

### Changes in Service Delivery

Table 2 compares essential procedures delivered by the participating doctors between baseline and endpoint and between intervention and control. Before application of JITIF, the proportion of patients who had received the listed service procedures was very low, ranging from 2.6% (for counseling symptoms relief) to 24.8% (for checking body signs). About 1 year’s implementation of JITIF resulted in substantial improvement in all these procedures, practiced over 88.5% for all the RTI and GTI patients on the intervention arm. The control versus intervention differences at endpoint were all statistically significant (*P*<.001), whereas no statistically significant difference was observed between patients on the two arms at the baseline. Although marginal increases were observed in the majority of the procedures from baseline to endpoint within the control group (eg, from 13.7% to 15.7% for performing auscultation; from 10.6% to 11.8% for telling diagnosis; and from 4.6% to 6.7% for discussing treatment alternatives), these changes were not significant (*P*=.51 to .98).

**Table 1:** Sociodemographic characteristics and symptomatic infections.

Participant group		Baseline			Endpoint			Overall *P* value
		Control (n=263), n (%)	Intervention (n=269), n (%)	*P* value	Control (n=254), n (%)	Intervention (n=262), n (%)	*P* value	
**Age**				.90			.81	.97
	≤30	52 (19.8)	53 (19.7)		50 (19.7)	54 (20.6)		
	31-40	51 (19.4)	58 (21.6)		49 (19.3)	57 (21.8)		
	41-50	53 (20.2)	59 (21.9)		51 (20.1)	55 (21.0)		
	51-60	52 (19.8)	47 (17.5)		49 (19.3)	50 (19.1)		
	≥61	55 (20.8)	52 (19.3)		55 (21.6)	46 (17.6)		
**Gender**				.65			.42	.63
	Male	86 (32.7)	83 (30.9)		75 (29.5)	86 (32.8)		
	Female	177(67.3)	186 (69.1)		179 (70.5)	176 (67.2)		
**Education**				.94			.73	.999
	Illiteracy	63 (24.0)	60 (22.3)		67 (26.4)	58 (22.1)		
	Primary school	72 (27.4)	71 (26.4)		61 (24.0)	68 (26.0)		
	Middle school	85 (32.3)	92 (34.2)		85 (33.5)	91 (34.7)		
	High school	43 (16.3)	46 (17.1)		41 (16.1)	45(17.2)		
**Clinically diagnosed infections**				.81			.70	.37
	RTIs^a^	200 (76.0)	207 (77.0)		207 (81.5)	210 (80.2)		
	GTIs^b^	63 (24.0)	62 (23.0)		47 (18.5)	52 (19.8)		

^a^RTIs: respiratory tract infections.

^b^GTIs: gastrointestinal tract infections.

### Changes in Patients’ Beliefs and Knowledge

Table 3 presents changes in beliefs and knowledge about infections and antibiotics among the patients. At baseline, most of the patients held no-objection even favoring (Yes or Not clear) views toward using antibiotics for common symptoms of RTIs or GTIs. Majority of them thought that antibiotics should generally be used for common cold (71.9% and 71.4% among the control and intervention groups at baseline, respectively), sore throat (84.8% and 85.1%), fever (70.0% and 69.9%), rhinorrhea (71.1% and 71.4%), indigestion (33.5% and 36.5%), acute gastroenteritis (87.8% and 88.5%), and diarrhea (63.1% and 63.9%). These percentages reduced by 6.0% (for beliefs in antibiotics use for sore throat) to 22.8% (for beliefs in antibiotics use for indigestion) as compared between control versus intervention, and almost all of the changes were statistically significant (*P*<.05) except that for sore throat (*P*=.08). The patients’ knowledge was also quite unfavorable at baseline. Those who were able to tell at least 1 specific side effect and measure for managing or preventing RTIs or GTIs accounted for less than 35.8%, 42.5%, and 46.8%, respectively, at baseline. These all witnessed significant increases at endpoint on the intervention arm, being 73.7%, 66.7%, and 69.2%, respectively.

### Changes in Antibiotics Prescription

Table 4 shows antibiotics prescription for patients between baseline and endpoint and between control and intervention groups. Put together, the proportion of symptomatic RTI or GTI patients who had been prescribed with antibiotics decreased from 88.0% at baseline to 62.3% at endpoint on the intervention arm (*P*<.001) but showed little change during the same period on the control arm, being 91.7% versus 89.7%, respectively (*P*=.48). This decrease was consistent for RTIs (from 87.1% to 64.3%, *P*<.001) and GTIs (from 94.7% to 52.4%, *P*<.001). By comparison, the reduction in oral antibiotics was substantially greater than that in intravenous antibiotics being from 60.5% to 42.3% versus from 62.5% to 12.8% for RTI and GTI patients.

**Table 2:** Practices of essential service procedures at baseline and endpoint.

Procedure		Baseline			Endpoint			Overall *P* value
		Control, n (%)	Intervention, n (%)	*P* value	Control, n (%)	Intervention, n (%)	*P* value	
**Asking history of previous treatment for the current illness**								
	Yes	52 (19.8)	54 (20.1)	.93	52 (20.5)	237 (90.5)	<.001	<.001
	No	211 (80.2)	215 (79.9)		202 (79.5)	25 (9.5)		
**Checking body signs (eg, swallow tonsils for RTIs or dehydration for GTIs)**								
	Yes	65 (24.7)	59 (21.9)	.45	63 (24.8)	236 (90.1)	<.001	<.001
	No	198 (75.3)	210 (78.1)		191 (75.2)	26 (9.9)		
**Measuring temperature**								
	Yes	62 (23.6)	57 (21.2)	.51	61 (24.0)	238 (90.8)	<.001	<.001
	No	201 (76.4)	212 (78.8)		193 (76.0)	24 (9.2)		
**Performing auscultation of the chest for RTIs^a^or the abdomen for GTIs^b^**								
	Yes	36 (13.7)	37 (13.8)	.98	40 (15.7)	232 (88.5)	<.001	<.001
	No	227 (86.3)	232 (86.2)		214 (84.3)	30 (12.2)		
**Telling diagnosis and disease trajectory**								
	Yes	28 (10.6)	27 (10.0)	.82	30 (11.8)	242 (92.4)	<.001	<.001
	No	235 (89.4)	242 (90.0)		224 (88.2)	20 (7.6)		
**Explaining potential causes of the illness**								
	Yes	17 (6.5)	15 (5.6)	.67	17 (6.7)	242 (92.4)	<.001	<.001
	No	246 (93.5)	254 (94.4)		237 (93.3)	20 (7.6)		
**Discussing treatment alternatives**								
	Yes	12 (4.6)	16 (5.9)	.47	17 (6.7)	247 (94.3)	<.001	<.001
	No	251 (95.4)	253 (94.1)		237 (93.3)	15 (5.7)		
**Educating drug intake if applicable**								
	Yes	32 (12.2)	31 (11.5)	.82	32 (12.6)	249 (95.0)	<.001	<.001
	No	231 (87.8)	238 (88.5)		222 (87.4)	13 (5.0)		
**Counseling symptoms relief**								
	Yes	8 (3.0)	7 (2.6)	.76	7 (2.8)	248 (94.7)	<.001	<.001
	No	255 (97.0)	262 (97.4)		247 (97.2)	14 (5.3)		
**Counseling infection prevention**								
	Yes	9 (3.4)	8 (3.0)	.77	8 (3.1)	241 (92.0)	<.001	<.001
	No	254 (96.6)	261 (97.0)		246 (96.9)	21 (8.0)		

^a^RTIs: respiratory tract infections.

^b^GTIs: gastrointestinal tract infections.

**Table 3:** Changes in patients’ beliefs and knowledge about antibiotics and infections.

Questionnaire item		Baseline			Endpoint			Overall *P* value
		Control, n (%)	Intervention, n (%)	*P* value	Control, n (%)	Intervention, n (%)	*P* value	
**Do you think antibiotics should generally be used for common cold?**								
	Yes or Not clear	189 (71.9)	192 (71.4)	.90	182 (71.7)	156 (59.5)	.004	.004
	No	74 (27.2)	77 (28.6)		72 (28.3)	106 (40.5)		
**Do you think antibiotics should be generally used for sore throat?**								
	Yes or Not clear	223 (84.8)	229 (85.1)	.91	213 (83.9)	204 (77.9)	.08	.03
	No	40 (15.2)	40 (14.9)		41 (16.1)	58 (22.1)		
**Do you think antibiotics should generally be used for fever?**								
	Yes or Not clear	184 (70.0)	188 (69.9)	.96	171 (67.3)	148 (56.5)	.01	.001
	No	79 (30.0)	81 (30.1)		83 (32.7)	114 (43.5)		
**Do you think antibiotics should generally be used for rhinorrhea?**								
	Yes or Not clear	187 (71.1)	192 (71.4)	.95	181 (71.3)	157 (59.9)	.01	.005
	No	76 (28.9)	77 (28.6)		73 (28.7)	105 (40.1)		
**Do you think antibiotics should generally be used for indigestion?**								
	Yes or Not clear	175 (66.5)	172 (63.9)	.53	153 (60.2)	98 (37.4)	<.001	<.001
	No	88 (33.5)	97 (36.1)		101 (39.8)	164 (62.6)		
**Do you think antibiotics should generally be used for acute gastroenteritis?**								
	Yes or Not clear	231 (87.8)	238 (88.5)	.82	221 (87.0)	210 (80.2)	.04	.008
	No	32 (12.2)	31 (11.5)		33 (13.0)	52 (19.8)		
**Do you think antibiotics should generally be used for diarrhea?**								
	Yes or Not clear	166 (63.1)	172 (63.9)	.84	158 (62.2)	130 (49.6)	.004	.001
	No	97 (36.9)	97 (36.1)		96 (37.8)	132 (50.4)		
**Can you name any specific side effects of antibiotics use?**								
	Yes	92 (35.0)	96 (35.7)	.87	91 (35.8)	193 (73.7)	.001	<.001
	No or Not clear	171 (65.0)	173 (64.3)		163 (64.2)	69 (26.3)		
**Can you name any specific measures for preventing or managing RTIs^a^?**								
	Yes	80 (40.0)	81 (39.1)	.86	88 (42.5)	140 (66.7)	<.001	<.001
	No or Not clear	119 (60.0)	126 (60.9)		119 (57.5)	70 (33.3)		
**Can you name any specific measures for preventing or managing GTIs^b^?**								
	Yes	30 (47.6)	29 (46.8)	.93	22 (46.8)	36 (69.2)	.02	.02
	No or Not clear	33 (52.4)	33 (53.2)		25 (53.2)	16 (30.8)		

^a^RTIs: respiratory tract infections.

^b^GTIs: gastrointestinal tract infections.

**Table 4:** Antibiotics prescription by baseline, endpoint, control, and intervention groups.

Antibiotics prescription		Baseline			Endpoint			Overall *P* value
		Control, n (%)	Intervention, n (%)	*P* value	Control, n (%)	Intervention, n (%)	*P* value	
**Antibiotics prescriptions for RTIs^a^**								
	Oral	97 (66.0)	122 (65.6)	.94	92 (60.5)	85 (42.3)	.001	<.001
	Intravenous	89 (53.0)	95 (54.0)	.89	85 (51.2)	71 (38.4)	.02	.003
	Any	159 (90.3)	176 (87.1)	.33	156 (89.7)	135 (64.3)	<.001	<.001
**Antibiotics prescriptions for GTIs^b^**								
	Oral	34 (68.0)	32 (64.0)	.67	25 (62.5)	6 (12.8)	<.001	<.001
	Intravenous	30 (60.0)	34 (58.6)	.88	21 (60.0)	16 (36.4)	.04	.03
	Any	50 (96.2)	54 (94.7)	.72	36 (90.0)	22 (52.4)	<.001	<.001
**Antibiotics prescriptions for RTIs and/or GTIs**								
	Oral	131 (66.5)	154 (65.3)	.79	117 (60.9)	91 (36.7)	<.001	<.001
	Intravenous	119 (54.6)	129 (55.1)	.91	106 (52.7)	87 (38.0)	.002	<.001
	Any	209 (91.7)	230 (88.8)	.29	192 (89.7)	157 (62.3)	<.001	<.001

^a^RTIs: respiratory tract infections.

^b^GTIs: gastrointestinal tract infections.

## Discussion

### Principal Findings

As mentioned earlier in the Introduction, many studies have been performed to determine the effectiveness of different types of interventions in promoting a more rational use of antibiotics at primary care settings, and multifaceted interventions have generally been more successful, especially if they employ SDM, involve peers, and benefit the practice as a whole [16]. Examples of such interventions include the Treat Antibiotics Responsibly, Guidance and Education Tool [17], the Stemming the Tide of Antibiotic Resistance [4], and the Nudging Guideline-Concordant Antibiotic Prescribing program [18]. These programs resulted in the reduction in inappropriate antibiotic prescribing by less than 20%. This study documented moderate to substantial changes, as a result of JITIF, in terms of: (1) prescribed antibiotics for RTI or GTI patients; (2) practice of essential clinical procedures; and (3) patients’ beliefs in and knowledge about antibiotics and infections. These changes are consistent with each other, and all point to an encouraging implication that the intervention is effective, at least in the short term. Most notably, the reduction in antibiotics use was about 10% greater than that of previous programs. This may be attributed partly to higher antibiotics prescription at baseline among our population.

Perhaps, the primary reason underlying the efficacy of this study may be that JITIF incorporates a number of theory- or evidence-based approaches in a synergetic way. The public commitment, for example, originated from an alternative behavior model holds that: (1) people place a high value on consistency and follow through with their public commitment to avoid disapproval by their peers and (2) publicly committing to a behavior prompts people to later justify that behavior and identify the behavior with their self-image, which may enhance personal dedication to performing that behavior [18,19]. Unlike other passive methods to improve quality of medical care such as financial incentives, public commitment features low cost and high sustainability. The inclusion of SDM was based on research findings that good level of SDM occurs only about 10% of the time [20] and that perceived pressure from patients has often been mentioned as a major reason for excessive antibiotics prescribing [21]. With options being clearly communicated to the patient and the patient’s preferences and expectations being explicitly sought, SDM should reduce irrational demand and expectations among the patients and correct misperceptions of patients’ needs among the doctors. As for the provision of service references, performance feedback, and Web-based SOPs, they were designed in accordance with planned behavior theory [22,23]. The theory states that attitude toward behavior, subjective norms, and perceived behavioral control, together shape an individual's behavioral intentions and practices. On the basis of authorized guidelines, the service references list positive behaviors for the doctors to practice (attitude), and the performance feedback tells the doctor that the majority of his or her peers are practicing to the references (subjective norm), while the Web-based SOPs provide easy ways in implementing positive behaviors and thus enhance the doctor’s sense of behavioral control.

The study also revealed useful information for better understanding routine health care for patients with symptomatic RTIs or GTIs at primary care settings in rural Anhui, China. Each of the procedures observed in this study (Table 2) has important implications for service quality. Researches showed that over one-third of the patients with symptomatic RTIs or GTIs had used self-obtained antibiotics before seeking help from village clinics [24]. So, there are reasons for village doctors to enquire and take into account prior treatment in planning current therapies for their patients. Similarly, checking body signs, measuring temperature, and performing auscultation helps doctors in reaching better understanding of the patients’ conditions and in gaining their trust and loyalty [25,26], whereas telling diagnosis and disease cause and trajectory reassures recovery within a short period (eg, 1-2 weeks) and reduces worries and, thus, demands for excessive treatment among the patients [27]. As for the remaining procedures, discussing treatment alternatives forms the key part of SDM which is, as mentioned above, beneficial in a number of ways; educating drug intake and symptom relief alleviates suffering and increases treatment compliance [28], whereas counseling infection reduces secondary infection (to family members) and reinfection of the patient himself or herself in the future [29]. However, these procedures were seldom practiced as evidenced by our observations at baseline on both the arms and at endpoint on the control arm (in less than 25% of the cases). Fortunately, JITIF seemed to work very well in leveraging delivery of these procedures. In short, the study indicates that: (1) the current care for patients with infections at rural primary care settings in China falls short in terms of most of the essential procedures, and thus there is a clear need to reverse the situation and (2) JITIF may be an easily applicable and effective approach in reaching this end.

### Strengths and Limitations

This study has both strength and weakness. Its strength originates from: (1) a packaged intervention consisting of cost-effective and sustainable approaches incorporated in a synergetic way and (2) an efficacy evaluation using a randomized controlled trial design that involved both baseline versus endpoint and control versus intervention comparisons. Perhaps, the biggest concern of the study may be observation-induced interferences on the practice behaviors. When being observed, the doctors may be more compliant to authorized guidelines [30]. To minimize such influences, the observation on the control and intervention arms used the same observers and identical protocol. However, doctors on the intervention arm were given detailed references, SOPs, and feedback, and thus they knew much better about what they were expected to do than those in the control group. Even so, the study findings suggest that JITIF may prove to be an effective intervention under closely observed or monitored conditions, and we are planning to leverage this study into a larger-scale randomized controlled trial that uses continuous artificial intelligence–based audio recording of the service encounters between village doctors and patients with infection symptoms. Another drawback of the study relates to nonblinded data collection, especially, rating of essential service procedures. The field data collectors may have given, due to various reasons, more positive ratings to intervention than the control groups since they knew the grouping, though the combination of the data quality control measures may have helped in keeping to a minimum. A third shortcoming concerns the use of antibiotics prescription as the primary measure in assessing JITIF efficacy. Given the prevalent use of antibiotics (as high as over 86% for symptomatic RTI or GTI patients), there are reasons to believe that JITIF helped in reducing excessive antibiotics prescription and thus is beneficial. However, less prescribed antibiotics may not necessarily mean better outcomes for all the patients [31]. And there is still a need of a further outcome-oriented evaluation, for example, linking JITIF with recovery from RTIs or GTIs and direct and indirect costs due to the infections. In addition, although our preliminary evaluation indicates that JITIF is quite effective as a whole, it is hard to tell what each of the individual ingredients of the package had contributed.

In conclusion, excessive use of antibiotics was very prevalent, and most essential service procedures for patients with symptomatic infections were not commonly practiced at primary care settings in rural Anhui, China. JITIF was effective in reducing antibiotic use and improving service procedures.

## Appendix

Multimedia Appendix 1 Commitment letter.

Multimedia Appendix 2 Sample patient takeaway information.

Multimedia Appendix 3 Sample screenshot of Web-based aid.

Multimedia Appendix 4 Checklist for observation of essential service procedures.

Multimedia Appendix 5 Additional information on web-based aid for just-in-time information and feedback (JITIF-WBA).

Multimedia Appendix 6 CONSORT E-HEALTH checklist (V 1.6.1).

## Abbreviations

ABR: antibiotic resistance
ABU: antibiotic use
EDI: eye and dental infection
GTI: gastrointestinal tract infection
JITIF: just-in-time information and feedback
PS: performance score
RTI: respiratory tract infection
SDM: shared decision-making
SI: skin infection
SOP: standard operation procedure
UTI: urinary tract infection
WBA: Web-based aid
