# Supplementary material for: Web-Based Just-in-Time Information and Feedback on Antibiotic Use for Village Doctors in Rural Anhui, China: Randomized Controlled Trial
Source: J Med Internet Res. 2018 Feb 14;20(2):e53. doi: 10.2196/jmir.8922 (PMC5830611; doi:10.2196/jmir.8922)
Supplement: Multimedia Appendix 1 [file jmir_v20i2e53_app1.pdf]

Annex 1: Commitment letter

Dear patients and your families,

As doctors, we hereby make our solemn commitments: 1) we will always adopt the best therapies for you; 2) we will try not to use unnecessary antibiotics (or “Xiaoyanyao”). The reasons why we make these commitments are as the following:

- Antibiotics (e.g., penicillin) are used to treat bacterial infections, but are not effective on diseases caused by virus;
- For non-bacterial infections, use of antibiotics not only has no efficacy, but also risks adverse reactions;
- Adverse reactions of antibiotics include harms to the liver, kidney, stomach, intestines and auditory function, rash, diarrhea, reproductive tract infections, and others;
- The more antibiotics are used, the higher the possibility of antibiotic resistance may occur; and treatment of the resistant bacterial infections can be very hard.
- Transfusion can not only increase efficacy of drugs, but also cause infections, transfusion reaction, air embolism, and others. It is also time-consuming and expensive. Oral administration is better than injection and in turn than intravenous transfusion.

It is very important to use antibiotics with caution. Antibiotics can only be administrated when one really needs it. Do not use antibiotics without the doctor’s permission and guidance, or otherwise it is unsafe and ineffective. Cough, sore throat, diarrhea and otitis media are not caused by bacteria in most cases, where there is no need to administer antibiotics. Doctors who often prescribe antibiotics are not accountable doctors.

Your health is our pursuit.

Name of signature doctor:

Date of signature:

Technically supported by Anhui Medical University and Anhui Provincial Hospital
