# Supplementary material for: Web-Based Just-in-Time Information and Feedback on Antibiotic Use for Village Doctors in Rural Anhui, China: Randomized Controlled Trial
Source: J Med Internet Res. 2018 Feb 14;20(2):e53. doi: 10.2196/jmir.8922 (PMC5830611; doi:10.2196/jmir.8922)
Supplement: Multimedia Appendix 2 [file jmir_v20i2e53_app2.pdf]

Annex 2 Sample patient takeaway information: respiratory tract infections

Patient Name__________

What symptoms do you have now?

- ☐ Nasal obstruction/running nose
- ☐ Cough
- ☐ Expectoration
- ☐ Sore throat
- ☐ Congestion/swelling of tonsils
- ☐ Fever
- ☐ Headache
- ☐ Earache, tinnitus and otorrhea
- ☐ Muscular stiffness
- ☐ Chest congestion/shortness of breath and dyspnea
- ☐ Rale in the lung
- ☐ Others________

☐ **What may be your current disease?**

- ☐ Upper respiratory infection
- ☐ Sphagitis
- ☐ Acute tonsillitis
- ☐ Acute otitis media
- ☐ Acute otitis externa
- ☐ Nasosinusitis
- ☐ Acute tracheitis/bronchitis
- ☐ Pneumonia
- ☐ Acute episode of chronic bronchitis
- ☐ Others__________

☐ **How long you may recover?**

- ☐ 1-2 days
- ☐ 3-4 days
- ☐ 5-6 days
- ☐ 1-2 weeks
- ☐ 3 weeks or longer
- ☐ Others_________

☐ **What may have caused your disease?**

- ☐ Virus
- ☐ Bacteria
- ☐ Fungus
- ☐ Parasites
- ☐ Others_________

☐ **What should you do with your current sickness?**

- ☐ Get some rest and drink more warm water
- ☐ Ventilate the room often
- ☐ Wear a mask when going out
- ☐ Go to crowded places less frequently
- ☐ Eat more vegetables and fruits
- ☐ Eat more nutrient fish, lean meat, eggs, etc.
- ☐ Do not smoke or drink
- ☐ Others________

☐ **Do you need antibiotics or "Xiaoyanyao"?**

- ☐ Antibiotics are effective to bacterial, Chlamydia and mycoplasma infections only, but ineffective to virus
- ☐ Most upper respiratory infections are caused by virus, so use of antibiotics will not help recovery more rapidly
- ☐ You don't need antibiotics or "Xiaoyanyao"
- ☐ You need antibiotics or "Xiaoyanyao"
- ☐ Antibiotics should be used with caution and only doctors can judge whether it is necessary or not
- ☐ Use of antibiotics causes resistance to them and thus makes it harder to treat the same infections next time
- ☐ If you get infected with drug-resistant bacteria, they can transmit to your families, especially children
- ☐ Antibiotics may harm human organs such as liver, kidney, stomach and intestines
- ☐ Antibiotics may disrupt normal flora in the digestive tract and so on and then cause malnutrition and others
- ☐ Use of antibiotics increase economic burden
- ☐ Antibiotics should be administered strictly according to the dosage and time on the prescription
- ☐ Others____

☐ **What should you do to protect you and your families from the same infection?**

- ☐ Do not smoke and drink less
- ☐ Avoid going to crowded places in flu seasons
- ☐ Avoid contacting with patients
- ☐ Wash hands often and keep personal hygiene
- ☐ Clean and ventilate your rooms regularly
- ☐ Eat more fruits and vegetables
- ☐ Do more exercises and physical activities
- ☐ Take vaccines against influenza
- ☐ Avoid over fatigue and keep spirits up
- ☐ Others_____________________________

☐ **When will you need to see a doctor again?**

- ☐ Not improved in 3-5 days
- ☐ Onset of serious headache and feel of nausea
- ☐ Onset of sleepiness, alalia and doze
- ☐ Onset of polypnea and blue lips
- ☐ Onset of dysphagia or drooling
- ☐ Onset of hemoptysis
- ☐ Onset of chest congestion, chest pain and dyspnea
- ☐ Others____

☐ 3

☐ **Doctor's Signature __________ Date of Visit_________**
