# Supplementary material for: Web-Based Just-in-Time Information and Feedback on Antibiotic Use for Village Doctors in Rural Anhui, China: Randomized Controlled Trial
Source: J Med Internet Res. 2018 Feb 14;20(2):e53. doi: 10.2196/jmir.8922 (PMC5830611; doi:10.2196/jmir.8922)
Supplement: Multimedia Appendix 3 [file jmir_v20i2e53_app3.pptx]

## Slide 1
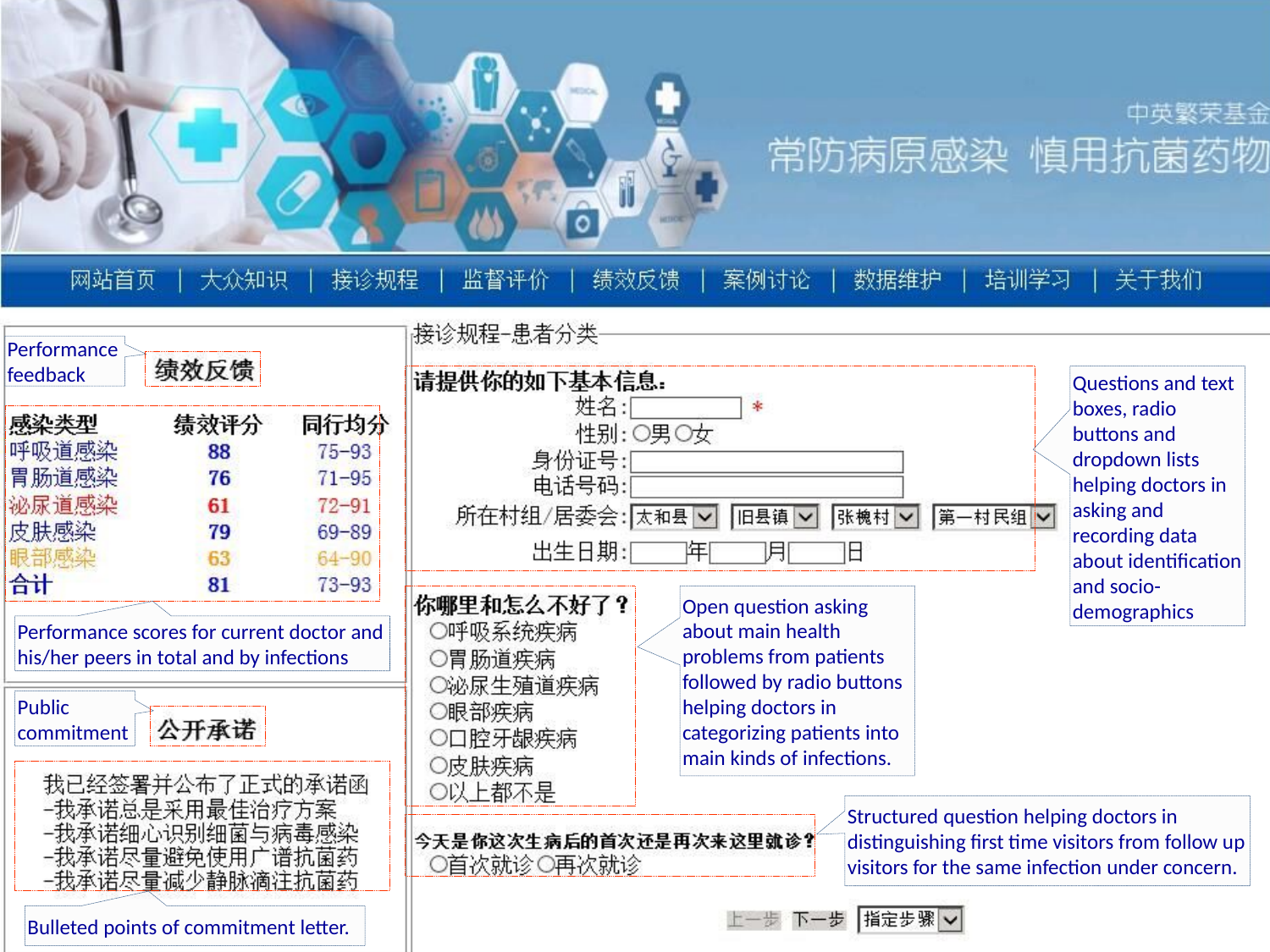

Performance feedback
Questions and text boxes, radio buttons and dropdown lists helping doctors in asking and recording data about identification and socio-demographics
Open question asking about main health problems from patients followed by radio buttons helping doctors in categorizing patients into main kinds of infections.
Performance scores for current doctor and his/her peers in total and by infections
Public commitment
Structured question helping doctors in distinguishing first time visitors from follow up visitors for the same infection under concern.
Bulleted points of commitment letter.
