# Supplementary material for: Web-Based Just-in-Time Information and Feedback on Antibiotic Use for Village Doctors in Rural Anhui, China: Randomized Controlled Trial
Source: J Med Internet Res. 2018 Feb 14;20(2):e53. doi: 10.2196/jmir.8922 (PMC5830611; doi:10.2196/jmir.8922)
Supplement: Multimedia Appendix 4 [file jmir_v20i2e53_app4.pdf]

Annex 4 Checklist for observation of essential service procedures

Number of patient encounter： |__|__|__|__|__|__|__|__|
Name of local village： county townships village group
Time point: |__|Baseline |__|Endpoint

P1: Asking history of previous treatment for the current illness

- ☐ Yes: if the doctors asked his/her patient of anything of above question
- ☐ No: otherwise.

P2: Checking body signs (e.g., swallow tonsils for RTIs or dehydration for GITs)

- ☐ Yes: if the doctor checked his/her patient for any potential body signs, e.g., swallow tonsils, dehydration
- ☐ No: otherwise.

P3: Measuring temperature

- ☐ Yes: if doctor measured his/her patient’s body temperature
- ☐ No: otherwise.

P4: Performing auscultation of the chest for RTIs or the abdomen for GTIs

- ☐ Yes: if the doctor performed auscultation of his/her patient’s chest or abdomen
- ☐ No: otherwise.

P5: Telling diagnosis and disease trajectory

- ☐ Yes: if the doctor told his/her patient anything about both the diagnosis and trajectory of the patient’s symptom
- ☐ No: otherwise.

P6: Explaining potential causes of the illness

- ☐ Yes: if the doctor explained anything about potential causes of the illness to his/her patient
- ☐ No: otherwise.

P7: Discussing treatment alternatives

- ☐ Yes: if the doctor discussed more than one treatment options with and asked any question about his/her patient’s opinions
- ☐ No: otherwise.

P8: Educating drug intake if applicable

- ☐ Yes: if the doctor had prescribed medicine(s) and told his/her patient anything about how to use the medicine(s)
- ☐ No: if the doctor had prescribed medicine(s) but did not tell his/her patient anything about how to use the medicine(s).
- ☐ Not applicable: if the doctor had not prescribe any medicine for his/her patient

P9: Counseling symptoms relief

- ☐ Yes: if the doctor taught his/her patient anything about how to manage the symptoms
- ☐ No: otherwise.

P10: Counseling infection prevention

- ☐ Yes: if the doctor taught his/her patient anything about how to prevent the same infection in the future both for himself/herself and his/her families
- ☐ No: otherwise.

Type of infection:

- ☐ RTIs
- ☐ GTIs

Signature of observer:

Date of observation: |__|__|-|__|__|-|__|__|(dd-mm-yy)
