# Supplementary material for: Web-Based Just-in-Time Information and Feedback on Antibiotic Use for Village Doctors in Rural Anhui, China: Randomized Controlled Trial
Source: J Med Internet Res. 2018 Feb 14;20(2):e53. doi: 10.2196/jmir.8922 (PMC5830611; doi:10.2196/jmir.8922)
Supplement: Multimedia Appendix 5 [file jmir_v20i2e53_app5.pdf]

Additional information on web-based aid for just-in-time information and feedback (JITIF-WBA)

Bug fixes, Downtimes, Content Changes

JITIF-WBA had been tested for over half years at multiple site clinics before commence of the current study. So, use of JITIF-WBA did not encounter any bugs during the study period. However, electricity cut-offs happened 2 times at 2 of the site clinics in which the village doctors resumed to traditional manual care for patients. Each of these power cut-offs lasted for less than half a day.

Computer / Internet literacy

The users of JITIF-WBA were village doctors. They have minimum knowledge and skills in using computers and web-based systems. In rural Anhui, China, all village clinics have personal computers and un-limited access to the internet. So, computer/internet literacy and facilities did not affect selection and randomization of participating clinics.

Display of institutional affiliations

Sponsored by the current project, JITIF-WBA was developed by a joint panel of the experts on care of infectious diseases, health services research and information technicians organized the current project team with Anhui Medical University. These were clearly conveyed to all participating village doctors on the intervention arm during the training workshop at the beginning of the project.

History/development process

Development of JITIF-WBA utilized Microsoft Visual Studio 2008 as platform, SQL Server 2008 as data management tool, C# as programming language and evolutionary prototyping as overall development approach in which design of initial type system followed by continuous refinement cycles. The whole process featured

dynamic interaction between development of practical and effective standardized operation procedures (SOPs) and web-based assistance for implementing these SOPs.

Initial SOPs behind JITIF-WBA derived through evidence and theory-based consensus. Firstly, our research team on antibiotics use conducted a systematic literature review of related guidelines, protocols, theories and research articles and then worked out a long list of proven intervention approaches and models. Secondly, an expert panel consisting of experienced doctors on care of infectious diseases, village doctors, epidemiologists and health services researchers conveyed for a consensus meeting and produced a short list of proven interventions from the long one via clarification, brainstorming and rounds of voting. Thirdly, our technical groups on antibiotics use and web program development worked together and translated the short list of intervention approaches and models into primitive SOPs. Fourthly, the expert panel gathered again and revised the primitive SOPs into initial practical ones.

The initial JITIF-WBA obtained above had been refined via repeated cycles of test-feedback- revision, at 3 village clinics in rural Anhui for a one year period.

Revisions and updating

The current evaluation of JITIF-WBA started from August 20, 2015) and an ended on September 30, 2016. During this time, no changes were made to the JITIF-WBA.

Replicability/ digital preservation

JITIF-WBA is accessible at http://210.45.98.43:8010. For sample screenshots of JITIF-WBA, please refer to Multimedia Appendix 3.

Access to application

Only the village doctors with the 12 village clinics on the intervention arm were allowed to use JITIF-WBA. They needed not pay for accessing the application. But each of the participating village doctors was asked to register a personal account and was only able to record and view service encounters with his/her patients.

Monitoring of use

JITIF-WBA provides step-by-step service procedures for patients with symptomatic infections. The participating village doctors were asked to use JITIF-WBA during each of the encounters with their patients of symptomatic infections. The logins

together with items (e.g., symptoms reported, medicine used) checked, selected or entered via the JITIF-WBA page were all automatically posted and preserved at the webmaster. These data were used to generate the content of the *Feedback Component* of JITIF as described in the main manuscript.

Qualitative feedback

Qualitative feedback from the participating village doctors and patients who had experienced the JITIF-WBA mediated healthcare was obtained via focus groups at the workshop for training intervention arm village doctors and via face-to-face interviews during the test and revision cycles as described above.

Ending of the trial

The trial started and ended as planned and after ending of the trial, all participating village doctors were denied, clearly stated during recruitment, access to the JITIF-WBA. This was because we planned to refine JITIF-WBA according to findings from the current trial.

Declaration of interests

JITIF-WBA was used for non-profit purpose and, if proved effective, will be made available free of charge to all village clinics and health authorities in China. Both the study team and the team who had developed JITIF-WBA are affiliated with School of Health Services Management, Anhui Medical University, China. But they belong to different group and are with different departments within the School.
