# Supplementary material for: Web-Based Just-in-Time Information and Feedback on Antibiotic Use for Village Doctors in Rural Anhui, China: Randomized Controlled Trial
Source: J Med Internet Res. 2018 Feb 14;20(2):e53. doi: 10.2196/jmir.8922 (PMC5830611; doi:10.2196/jmir.8922)
Supplement: Multimedia Appendix 6 [file jmir_v20i2e53_app6.pdf]

CONSORT-EHEALTH checklist (V.1.6.1): Information to include when reporting ehealth/mhealth trials (web-based/Internet-based intervention and decision aids, but also social media, serious games, DVDs, mobile applications, certain telehealth applications)

Do you feel items are missing/unclear/unnecessary? Please comment at http://tinyurl.com/consort-ehealth-v1-5

If you are working on a manuscript submission, please fill in this checklist electronically at
http://tinyurl.com/consort-ehealth-v1-6

Section/Topic	Item No.	CONSORT* Checklist Item	EHEALTH Extensions (additions to, or clarification of the CONSORT item)	*Importance*
**TITLE & ABSTRACT**	1a	Identification as a randomized trial in the title	i) **Identify the mode of delivery in the title**. Preferably use “web-based” and/or “mobile” and/or “electronic game” in the title. Avoid ambiguous terms like “online”, “virtual”, “interactive”. Use “Internet-based” only if Intervention includes non-web-based Internet components (e.g., email), use “computer-based” or “electronic” only if offline products are used. Use “virtual” only in the context of “virtual reality” (3-D worlds). Use “online” only in the context of “online support groups”. Complement or substitute product names with broader terms for the class of products (such as “mobile” or “smart phone” instead of “iphone”), especially if the application runs on different platforms.	*Essential*<br>Yes
			ii) Mention **non-web-based components or important co-interventions in the title**, if any (e.g., “with telephone support”).	*Highly Recommended*
			iii) Mention **primary condition or target group in the title**, if any (e.g., “for children with Type I Diabetes”)<br>Example: *A Web-based and Mobile Intervention with Telephone Support for Children with Type I Diabetes: Randomized Controlled Trial*	*Essential*<br>Yes
	1b	Structured summary of trial design, methods, results, and conclusions<br>NPT** extension: Description of experimental treatment,	Methods (in Abstract):<br>i) Mention **key features/functionalities/components of the intervention and comparator in the abstract**. If possible, also mention theories and principles used for designing the site. Keep in mind the needs of systematic reviewers and indexers by including important synonyms.	*Essential*<br>Yes

comparator, care providers, centers, and blinding status	(Note: Only report in the abstract what the main paper is reporting. If this information is missing from the main body of text, consider adding it)	
	ii) Clarify the **level of human involvement in the abstract**, e.g., use phrases like "fully automated" vs. "therapist/nurse/care provider/physician-assisted" (mention number and expertise of providers involved, if any). (Note: Only report in the abstract what the main paper is reporting. If this information is missing from the main body of text, consider adding it)	*Highly Recommended*<br>Yes
	iii) **Open vs. closed, web-based (self-assessment) vs. face-to-face assessments in abstract:** Mention how participants were recruited (online vs. offline), e.g., from an open access website or from a clinic or a closed online user group (closed usergroup trial), and clarify if this was a purely web-based trial, or there were face-to-face components (as part of the intervention or for assessment). Clearly say if outcomes were *self-assessed* through questionnaires (as common in web-based trials). Note: In traditional offline trials, an open trial (open-label trial) is a type of clinical trial in which both the researchers and participants know which treatment is being administered. To avoid confusion, use "blinded" or "unblinded" to indicated the level of blinding instead of "open", as "open" in web-based trials usually refers to "open access" (i.e. participants can self-enrol) (Note: Only report in the abstract what the main paper is reporting. If this information is missing from the main body of text, consider adding it)	*Highly Recommended*<br>Yes
	iv) **Results in abstract must contain use data**: Report number of participants enrolled/assessed in each group, the *use/uptake* of the intervention (e.g., attrition/adherence metrics, use over time, number of logins etc.), in addition to primary/secondary outcomes. (Note: Only report in the abstract what the main paper is reporting. If this information is missing from the main body of text, consider adding it)	*Highly recommended*<br>Yes

			v) **Conclusions/Discussions in abstract for negative trials**: Discuss the primary outcome - if the trial is negative (primary outcome not changed), and the intervention was not used, discuss whether negative results are attributable to lack of uptake and discuss reasons.	*Highly Recommended*<br>Yes
**INTRODUCTION**<br>Background and objectives	2a	Scientific background and explanation of rationale	i) Describe the **problem and the type of system/solution** that is object of the study: intended as stand-alone intervention vs. incorporated in broader health care program? [1] Intended for a particular patient population? [1] Goals of the intervention, e.g., being more cost-effective to other interventions [1], replace or complement other solutions? (Note: Details about the intervention are provided in "Methods" under 5)	*Essential*<br>Yes
			ii) **Scientific background, rationale:** What is known about the (type of) system that is the object of the study (be sure to discuss the use of similar systems for other conditions/diagnoses, if appropriate), motivation for the study, i.e., what are the reasons for and what is the context for this specific study, from which stakeholder viewpoint is the study performed, potential impact of findings [2]. Briefly justify the choice of the comparator.	*Essential*<br>Yes
	2b	Specific objectives or hypotheses	*No EHEALTH-specific additions here*<br>(note: Contrary to STARE-HI we do not recommend to mention IRB approval in this section - JMIR and other journals typically recommend this as a subheading under "methods". CONSORT-EHEALTH has a separate item for ethical considerations)	Yes
**METHODS**<br>Trial design	3a	Description of trial design (such as parallel, factorial) including allocation ratio	*No EHEALTH-specific additions here*	Yes
	3b	Important changes to methods after trial commencement (such as eligibility criteria), with reasons	*i) **Bug fixes, Downtimes, Content Changes:** ehealth systems are often dynamic systems. A description of changes to methods therefore also includes important changes made on the intervention or comparator during the trial (e.g., major bug fixes or changes in the functionality or content) (5-iii) and other "unexpected events" that may have influenced study design*	*Highly Recommended*<br>Yes

			such as staff changes, system failures/downtimes, etc. [2].	
Participants	4a	Eligibility criteria for participants	*i) **Computer / Internet literacy** is often an implicit "de facto" eligibility criterion - this should be explicitly clarified [1].*	*Highly Recommended*<br>Yes
			*ii) **Open vs. closed, web-based vs. face-to-face assessments:** Mention how participants were recruited (online vs. offline), e.g., from an open access website or from a clinic, and clarify if this was a purely web-based trial, or there were face-to-face components (as part of the intervention or for assessment), i.e., to what degree the study team got to know the participant. In online-only trials, clarify if participants were quasi-anonymous and whether having multiple identities was possible or whether technical or logistical measures (e.g., cookies, email confirmation, phone calls) were used to detect/prevent these.*	*Essential*<br>Yes
			*iii) **Information given during recruitment**. Specify how participants were briefed for recruitment and in the informed consent procedures (e.g., publish the informed consent documentation as appendix, see also item X26), as this information may have an effect on user self-selection, user expectation and may also bias results.*	*Highly Recommended*<br>NA
	4b	Settings and locations where the data were collected	*i) Clearly **report if outcomes were (self-)assessed through online questionnaires** (as common in web-based trials) or otherwise.*	*Essential*<br>Yes
			*ii) **Report how institutional affiliations are displayed** to potential participants [on ehealth media], as affiliations with prestigious hospitals or universities may affect volunteer rates, use, and reactions with regards to an intervention" [1].(Not a required item – describe only if this may bias results)*	*Recommended*<br>Yes
Interventions	5	The interventions for each group with sufficient details to allow replication, including how and when they were actually	*i) **Mention names, credential, affiliations of the developers, sponsors, and owners** [6] (if authors/evaluators are owners or developer of the software, this needs to be declared in a "Conflict of interest" section or mentioned elsewhere in the manuscript).*	*Highly Recommended*<br>Yes

administered	*ii) **Describe the history/development process** of the application and previous formative evaluations (e.g., focus groups, usability testing), as these will have an impact on adoption/use rates and help with interpreting results.*	*Highly Recommended*<br>Yes
	*iii) **Revisions and updating**. Clearly mention the date and/or version number of the application/intervention (and comparator, if applicable) evaluated, or describe whether the intervention underwent major changes during the evaluation process, or whether the development and/or content was "frozen" during the trial. Describe dynamic components such as news feeds or changing content which may have an impact on the replicability of the intervention (for unexpected events see item 3b).*	*Highly Recommended*<br>Yes
	*iv) Provide information on **quality assurance methods** to ensure accuracy and quality of information provided [1], if applicable.*	*Highly Recommended*<br>NA
	*v) **Ensure replicability by publishing the source code, and/or providing screenshots/screen-capture video, and/or providing flowcharts of the algorithms used.** Replicability (i.e., other researchers should in principle be able to replicate the study) is a hallmark of scientific reporting.*	*Highly Recommended*<br>Yes
	*vi) **Digital preservation**: Provide the URL of the application, but as the intervention is likely to change or disappear over the course of the years; also make sure the intervention is archived (Internet Archive, webcitation.org, and/or publishing the source code or screenshots/videos alongside the article). As pages behind login screens cannot be archived, consider creating demo pages which are accessible without login.*	*Highly Recommended*<br>Yes
	*vii) **Access**: Describe how participants accessed the application, in what setting/context, if they had to pay (or were paid) or not, whether they had to be a member of specific group. If known, describe how participants obtained "access to the platform and Internet" [1]. To ensure access for editors/reviewers/readers, consider to provide a "backdoor" login account or demo mode for reviewers/readers to explore the application (also important for archiving purposes, see vi).*	*Essential*<br>Yes

	viii) Describe ***mode of delivery, features/functionalities/components of the intervention and comparator, and the theoretical framework*** *[6] used to design them (instructional strategy [1], behaviour change techniques, persuasive features, etc., see e.g., [7, 8] for terminology). This includes an in-depth description of the content (including where it is coming from and who developed it) [1], "whether [and how] it is tailored to individual circumstances and allows users to track their progress and receive feedback" [6]. This also includes a description of communication delivery channels and – if computer-mediated communication is a component – whether communication was synchronous or asynchronous [6]. It also includes information on presentation strategies [1], including page design principles, average amount of text on pages, presence of hyperlinks to other resources etc. [1].*	*Essential*<br>Yes
	ix) ***Describe use parameters*** *(e.g., intended "doses" and optimal timing for use) [1]. Clarify what instructions or recommendations were given to the user, e.g., regarding timing, frequency, heaviness of use [1], if any, or was the intervention used ad libitum.*	*Highly Recommended*<br>Yes
	x) ***Clarify the level of human involvement*** *(care providers or health professionals, also technical assistance) in the e-intervention or as co-intervention. Detail number and expertise of professionals involved, if any, as well as "type of assistance offered, the timing and frequency of the support, how it is initiated, and the medium by which the assistance is delivered" [6]. It may be necessary to distinguish between the level of human involvement required for the trial, and the level of human involvement required for a routine application outside of a RCT setting (discuss under item 21 – generalizability).*	*Highly recommended*<br>Yes
	xi) ***Report any prompts/reminders used:*** *Clarify if there were prompts (letters, emails, phone calls, SMS) to use the application, what triggered them, frequency, etc. [1]. It may be necessary to distinguish between the level of prompts/reminders required for the trial, and the level of prompts/reminders for a routine application outside of a RCT setting (discuss under item 21 – generalizability).*	*Essential*<br>NA

			xii) ***Describe any co-interventions (incl. training/support)****: Clearly state any "interventions that are provided in addition to the targeted eHealth intervention" [1], as ehealth intervention may not be designed as stand-alone intervention. This includes training sessions and support [1]. It may be necessary to distinguish between the level of training required for the trial, and the level of training for a routine application outside of a RCT setting (discuss under item 21 – generalizability.*	*Essential*<br>Yes
Outcomes	6a	Completely defined pre-specified primary and secondary outcome measures, including how and when they were assessed	*i) If outcomes were obtained through* ***online questionnaires, describe if they were validated for online use [6] and apply CHERRIES items to describe how the questionnaires were designed/deployed*** *[9].*	*Highly Recommended*<br>NA
			ii) ***Describe whether and how "use" (including intensity of use/dosage) was defined/measured/monitored*** *(logins, logfile analysis, etc.). Use/adoption metrics are important process outcomes that should be reported in any ehealth trial.*	*Highly Recommended*<br>Yes
			iii) Describe ***whether, how, and when qualitative feedback was obtained from participants*** *(e.g., through emails, feedback forms, interviews, focus groups).*	*Highly Recommended*<br>Yes
	6b	Any changes to trial outcomes after the trial commenced, with reasons	*No EHEALTH-specific additions here*	Yes
Sample size	7a	How sample size was determined NPT: When applicable, details of whether and how the clustering by care provides or centers was addressed	*i)* ***Describe whether and how expected attrition was taken into account when calculating the sample size***	*Highly Recommended*<br>NA
	7b	When applicable, explanation of any interim analyses and stopping guidelines	*No EHEALTH-specific additions here*	NA
Randomisation:				

Sequence generation	8a	Method used to generate the random allocation sequence<br>NPT: When applicable, how care providers were allocated to each trial group	*No EHEALTH-specific additions here*	Yes
	8b	Type of randomisation; details of any restriction (such as blocking and block size)	*No EHEALTH-specific additions here*	Yes
Allocation concealment mechanism	9	Mechanism used to implement the random allocation sequence (such as sequentially numbered containers), describing any steps taken to conceal the sequence until interventions were assigned	*No EHEALTH-specific additions here*	Yes
Implementation	10	Who generated the random allocation sequence, who enrolled participants, and who assigned participants to interventions	*No EHEALTH-specific additions here*	Yes
Blinding	11a	If done, who was blinded after assignment to interventions (for example, participants, care providers, those assessing outcomes) and how<br>NPT: Whether or not administering co-interventions were blinded to group assignment	*i)* ***Specify who was blinded, and who wasn't***. *Usually, in web-based trials it is not possible to blind the participants [1, 3] (this should be clearly acknowledged), but it may be possible to blind outcome assessors, those doing data analysis or those administering co-interventions (if any).*<br><br>*ii) Informed consent procedures (4a-ii) can create biases and certain expectations -* ***discuss e.g., whether participants knew which intervention was the "intervention of interest" and which one was the "comparator".***	*Essential*<br>Yes<br><br>*Highly Recommended*<br>Yes
	11b	If relevant, description of the similarity of interventions	*(this item is usually not relevant for ehealth trials as it refers to similarity of a placebo or sham intervention to a active medication/intervention)*	NA

Statistical methods	12a	Statistical methods used to compare groups for primary and secondary outcomes<br>NPT: When applicable, details of whether and how the clustering by care providers or centers was addressed	*i) **Imputation techniques to deal with attrition / missing values**: Not all participants will use the intervention/comparator as intended and attrition is typically high in ehealth trials. Specify how participants who did not use the application or dropped out from the trial were treated in the statistical analysis (a complete case analysis is strongly discouraged, and simple imputation techniques such as LOCF may also be problematic [4]).*	*Essential*<br>Yes
	12b	Methods for additional analyses, such as subgroup analyses and adjusted analyses	*No EHEALTH-specific additions here*	Yes
Ethics & Informed Consent	X26	*(not a CONSORT item)*	*i) **Comment on ethics committee approval.***	*Highly Recommended*
			*ii) **Outline informed consent procedures** e.g., if consent was obtained offline or online (how? Checkbox, etc.?), and what information was provided (see 4a-ii). See [6] for some items to be included in informed consent documents.*	*Highly Recommended*<br>Yes
			*iii) **Safety and security procedures**, incl. privacy considerations, and "any steps taken to reduce the likelihood or detection of harm (e.g., education and training, availability of a hotline)" [1].*	*Highly Recommended*<br>Yes
**RESULTS**<br>Participant flow (a diagram is strongly recommended)	13a	For each group, the numbers of participants who were randomly assigned, received intended treatment, and were analysed for the primary outcome<br>NPT: The number of care providers or centers performing the intervention in each group and the number of patients treated by each care provider in each center	*No EHEALTH-specific additions here*	Yes

	13b	For each group, losses and exclusions after randomisation, together with reasons	i) Strongly recommended: An **attrition diagram** (e.g., proportion of participants still logging in or using the intervention/comparator in each group plotted over time, similar to a survival curve) [5] or other figures or tables demonstrating usage/dose/engagement.	*Highly Recommended*<br>Yes
Recruitment	14a	Dates defining the periods of recruitment and follow-up	i) **Indicate if critical "secular events" [1] fell into the study period**, e.g., significant changes in Internet resources available or "changes in computer hardware or Internet delivery resources" [1].	*Highly Recommended*<br>No
	14b	Why the trial ended or was stopped [early]	*No EHEALTH-specific additions here*	Yes
Baseline data	15	A table showing baseline demographic and clinical characteristics for each group<br>NPT: When applicable, a description of care providers (case volume, qualification, expertise, etc.) and centers (volume) in each group	i) In ehealth trials it is particularly important to **report demographics associated with digital divide issues**, such as age, education, gender, social-economic status, computer/Internet/ehealth literacy of the participants, if known.	*Essential*<br>Yes
Numbers analysed	16	For each group, number of participants (denominator) included in each analysis and whether the analysis was by original assigned groups	i) **Report multiple "denominators" and provide definitions**: Report N's (and effect sizes) "across a range of study participation [and use] thresholds" [1], e.g., N exposed, N consented, N used more than x times, N used more than y weeks, N participants "used" the intervention/comparator at specific pre-defined time points of interest (in absolute and relative numbers per group). Always clearly define "use" of the intervention.	*Essential*<br>Yes
			ii) **Primary analysis should be intent-to-treat**; secondary analyses could include comparing only "users", with the appropriate caveats that this is no longer a randomized sample (see 18-i).	*Highly Recommended*<br>NA
Outcomes and estimation	17a	For each primary and secondary outcome, results for each group, and the estimated effect size and its precision (such as 95% confidence interval)	i) In addition to primary/secondary (clinical) outcomes, the **presentation of process outcomes such as metrics of use and intensity of use** (dose, exposure) and their operational definitions is critical. This does not only refer to metrics of attrition (13-b) (often a binary variable), but also to more continuous exposure metrics such as "average session length". These must be accompanied by a technical description how a	*Highly Recommended*<br>Yes

			metric like a "session" is defined (e.g., timeout after idle time) [1] (report under item 6a).	
	17b	For binary outcomes, presentation of both absolute and relative effect sizes is recommended	*No EHEALTH-specific additions here*	Yes
Ancillary analyses	18	Results of any other analyses performed, including subgroup analyses and adjusted analyses, distinguishing pre-specified from exploratory	i) A **subgroup analysis of comparing only users** is not uncommon in ehealth trials, but if done it must be stressed that this is a self-selected sample and no longer an unbiased sample from a randomized trial (see 16-iii).	*Highly Recommended* Yes
Harms	19	All important harms or unintended effects in each group (for specific guidance see CONSORT for harms)	i) **Include privacy breaches, technical problems**. This does not only include physical "harm" to participants, but also incidents such as perceived or real privacy breaches [1], technical problems, and other unexpected/unintended incidents. "Unintended effects" also includes unintended *positive* effects [2].	*Highly Recommended* NA
			ii) **Include qualitative feedback from participants or observations from staff/researchers**, if available, on strengths and shortcomings of the application, especially if they point to unintended/unexpected effects or uses. This includes (if available) reasons for why people did or did not use the application as intended by the developers.	*Highly Recommended* NA
Interpretation/ Principal Findings	22	Interpretation consistent with results, balancing benefits and harms, and considering other relevant evidence<br>NPT: In addition, take into account the choice of the comparator, lack of or partial blinding, and unequal expertise of care providers or centers in each group	i) **Restate study questions and summarize the answers suggested by the data [2], starting with primary outcomes and process outcomes (use).**	*Essential* Yes
			ii) **Highlight unanswered new questions, suggest future research** [2].	*Highly Recommended* Yes

**DISCUSSION** Limitations	20	Trial limitations, addressing sources of potential bias, imprecision, and, if relevant, multiplicity of analyses	i) **Typical limitations in ehealth trials**: Participants in ehealth trials are rarely blinded. Ehealth trials often look at a multiplicity of outcomes, increasing risk for a Type I error. Discuss biases due to non-use of the intervention/usability issues, biases through informed consent procedures, unexpected events.	*Essential* Yes
Generalisability	21	Generalisability (external validity, applicability) of the trial findings NPT: External validity of the trial findings according to the intervention, comparators, patients, and care providers or centers involved in the trial	i) **Generalizability to other populations:** In particular, discuss generalizability to a general *Internet* population, outside of a RCT setting, and general patient population, including applicability of the study results for other organizations [2].	*Highly Recommended* Yes
			ii) **Discuss if there were elements in the RCT that would be different in a routine application setting** (e.g., prompts/reminders, more human involvement, training sessions or other co-interventions) and what impact the omission of these elements could have on use, adoption, or outcomes if the intervention is applied outside of a RCT setting.	*Highly Recommended* Yes
**OTHER INFORMATION**				
Registration	23	Registration number and name of trial registry	*No EHEALTH-specific additions here*	NA
Protocol	24	Where the full trial protocol can be accessed, if available	*No EHEALTH-specific additions here*	NA
Funding	25	Sources of funding and other support (such as supply of drugs), role of funders	*No EHEALTH-specific additions here*	Yes
Competing interests	X27	*(not a CONSORT item)*	i) In addition to the usual declaration of interests (financial or otherwise), also **state the "relation of the study team towards the system being evaluated"** [2], i.e., state if the authors/evaluators are distinct from or identical with the developers/sponsors of the intervention.	*Highly Recommended* Yes

* CONSORT = Consolidated Standards of Reporting Trials [10]

** NPT = non pharmacological treatment (CONSORT extension) [11]

References

1. Baker TB, Gustafson DH, Shaw B, Hawkins R, Pingree S, Roberts L, Strecher V. Relevance of CONSORT reporting criteria for research on eHealth interventions. Patient Educ Couns. 2010 Dec;81 Suppl:S77-86
2. Talmon J, Ammenwerth E, Brender J, de Keizer N, Nykänen P, Rigby M. STARE-HI--Statement on reporting of evaluation studies in Health Informatics. Int J Med Inform. 2009 Jan;78(1):1-9. Epub 2008 Oct 18.
3. Eysenbach G. Issues in evaluating health websites in an Internet-based randomized controlled trial. J Med Internet Res 2002;4(3):e17
4. Blankers M, Koeter MWJ, Schippers GM. Missing Data Approaches in eHealth Research: Simulation Study and a Tutorial for Nonmathematically Inclined Researchers. J Med Internet Res 2010;12(5):e54
5. Eysenbach G. The law of attrition. J Med Internet Res 2005;7(1):e11
6. Proudfoot et al. Establishing Guidelines for Executing and Reporting Internet Intervention Research. Cognitive Behaviour Therapy (forthcoming)
7. Webb TL, Joseph J, Yardley L, Michie S. Using the Internet to Promote Health Behavior Change: A Systematic Review and Meta-analysis of the Impact of Theoretical Basis, Use of Behavior Change Techniques, and Mode of Delivery on Efficacy. J Med Internet Res 2010;12(1):e4
8. Cugelman B, Thelwall M, Dawes P. Online Interventions for Social Marketing Health Behavior Change Campaigns: A Meta-Analysis of Psychological Architectures and Adherence Factors. J Med Internet Res 2011;13(1):e17
9. Eysenbach G. Improving the Quality of Web Surveys: The Checklist for Reporting Results of Internet E-Surveys (CHERRIES). J Med Internet Res 2004;6(3):e34
10. Schulz KF, Altman DG, Moher D, for the CONSORT Group (2010) CONSORT 2010 Statement: Updated Guidelines for Reporting Parallel Group Randomised Trials. PLoS Med 7(3): e1000251
11. Boutron I, Moher D, Altman DG, Schulz K, Ravaud P, for the CONSORT group. Extending the CONSORT Statement to randomized trials of nonpharmacologic treatment: explanation and elaboration. Ann Intern Med. 2008:295-309

Note: Yes in blue stands for the corresponding item has been addressed in our manuscript or its multimedia appendixes; while NA in blue denotes the corresponding item does not apply to this study.
